# Distribution and Metabolism of Bt-Cry1Ac Toxin in Tissues and Organs of the Cotton Bollworm, *Helicoverpa armigera*

**DOI:** 10.3390/toxins8070212

**Published:** 2016-07-07

**Authors:** Zhuoya Zhao, Yunhe Li, Yutao Xiao, Abid Ali, Khalid Hussain Dhiloo, Wenbo Chen, Kongming Wu

**Affiliations:** 1 The State Key Laboratory for Biology of Plant Diseases and Insect Pests, Institute of Plant Protection, Chinese Academy of Agricultural Sciences, West Yuanmingyuan Road, Beijing 100193, China; zhaozhuoyavip@163.com (Z.Z.); yunheli@ippcaas.cn (Y.L.); xiao20020757@163.com (Y.X.); abid_ento74@yahoo.com (A.A.); khdhiloo@yahoo.com (K.H.D.); wenbochenkissy@163.com (W.C.); 2 Department of Entomology, University of Agriculture, Faisalabad 38000, Pakistan; 3 Department of Entomology, Faculty of Crop Protection, Sindh Agriculture University, Tandojam 70060, Pakistan

**Keywords:** Cry1Ac, *Helicoverpa armigera*, tissue distribution, toxin metabolism

## Abstract

Crystal (Cry) proteins derived from *Bacillus thuringiensis* (*Bt*) have been widely used in transgenic crops due to their toxicity against insect pests. However, the distribution and metabolism of these toxins in insect tissues and organs have remained obscure because the target insects do not ingest much toxin. In this study, several Cry1Ac-resistant strains of *Helicoverpa armigera*, fed artificial diets containing high doses of Cry1Ac toxin, were used to investigate the distribution and metabolism of Cry1Ac in their bodies. Cry1Ac was only detected in larvae, not in pupae or adults. Also, Cry1Ac passed through the midgut into other tissues, such as the hemolymph and fat body, but did not reach the larval integument. Metabolic tests revealed that Cry1Ac degraded most rapidly in the fat body, followed by the hemolymph, peritrophic membrane and its contents. The toxin was metabolized slowly in the midgut, but was degraded in all locations within 48 h. These findings will improve understanding of the functional mechanism of Bt toxins in target insects and the biotransfer and the bioaccumulation of Bt toxins in arthropod food webs in the Bt crop ecosystem.

## 1. Introduction

*Bacillus thuringiensis* (*Bt*) is a gram-positive, entomopathogenic, sporulating bacterium, which produces crystal (Cry) proteins or inclusion bodies that have selective insecticidal toxicity against different groups of insect pests [1,2,3]. Microbial preparations containing Cry protein (and bacterial cells and endospores) have been applied as foliar sprays in agricultural settings for several decades [4]. In the last decade, insecticidal crystal proteins (ICPs) encoded by the *Bt* gene have been introduced into many crops, making them resistant to lepidopteran or coleopteran pests [4,5,6]. In China, Bt cotton has been commercialized to control *Helicoverpa armigera* since 1997, and the pest population has greatly declined [7,8,9].

*H. armigera* is the most serious insect pest of cotton [10]. Several models have been proposed to explain the functional mechanism of the Cry protein against target insects. The most widely accepted model proposed that, the proteolytically activated toxins bind to specific receptors in the midgut epithelial cells, subsequently creating pores in the cell membrane and eventually killing the insect [11,12,13,14]. Investigating the distribution of the Cry protein in *H. armigera* may facilitate a better understanding of the mechanisms responsible for the insecticidal toxicity of the Cry protein.

In earlier studies, researchers mostly focused on the level of Cry toxin within the whole body of different insects [15,16,17]. In studies on the distribution of Cry1Ab in the tissues of *H. armigera* and *Mythimna unipuncta*, Cry toxins were detected in the midgut and hemolymph [18,19]. In *Spodoptera littoralis* analyzed at different developmental stages, the Cry toxins were found in larvae but not in adults [20]. However, to our knowledge, no study has investigated the metabolism of Bt toxins in insect larval tissues.

The lack of detection of Bt toxin in other tissues and the unknown status of toxin metabolism in larval tissues of *H. armigera* are probably a consequence of the high susceptibility of the insects to Bt toxin; they cannot survive high doses of the toxin. In the current study, several Cry1Ac-resistant strains of *H. armigera* were used, which can survive high doses of Cry1Ac toxin, thus allowing the study of the distribution and metabolism of Cry1Ac toxin in the larval tissues of *H. armigera*.

## 2. Results

### 2.1. Concentrations of Cry1Ac Toxin in H. armigera Bodies at Different Developmental Stages

**Figure 1:**
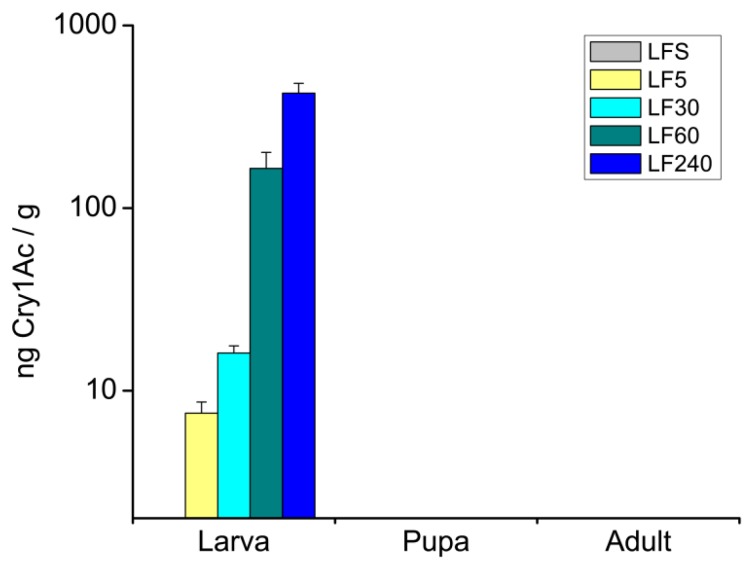
Mean concentrations (± SE) of Cry1Ac toxin (ng Cry1Ac/g insects, *n* = 5) in *H. armigera* larvae, pupae and adults. Susceptible strain LFS (Langfang susceptible) was reared on an artificial diet without Bt; Bt-resistant strains LF5, LF30, LF60 and LF240 were fed an artificial diet containing 5.00, 30.00, 60.00 or 240.00 μg/g of Cry1Ac, respectively.

ELISA (Enzyme-Linked Immunosorbent Assay) assays showed that larvae of all resistant strains of *H. armigera* contained Cry1Ac toxin when fed artificial diets containing 5.00 μg/g to 240.00 μg/g Cry1Ac, and the toxin level in the larvae increased as the toxin concentrations in the diet increased. However, the toxin was not detected in pupae or adults (Figure 1).

### 2.2. Tissue Distribution of Cry1Ac Toxin in H. armigera Larvae

**Figure 2:**
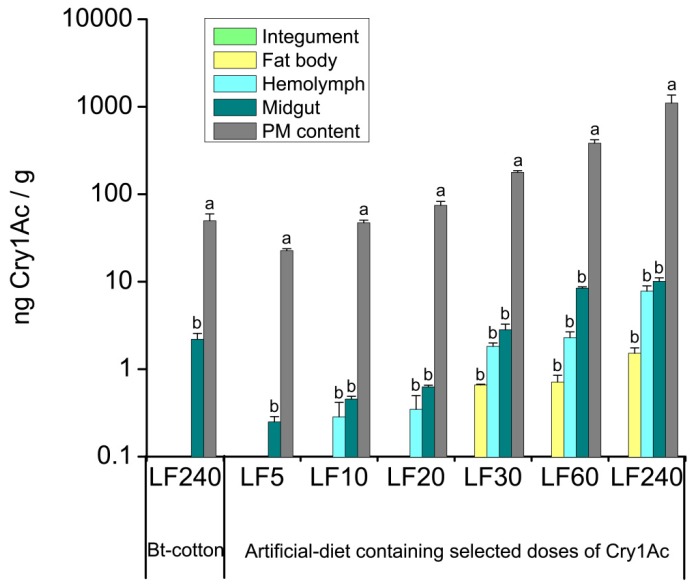
Enzyme-Linked Immunosorbent Assay (ELISA) results of Cry1Ac toxin levels in different tissues of larvae of resistant strains of *H. armigera*. Mean concentrations (± SE) of Cry1Ac toxin (ng Cry1Ac/g insects, *n* = 6) in the integument, fat body, hemolymph, midgut, peritrophic membrane and its contents (PM content) of LF240 strain larvae fed on Bt-cotton, and Bt-resistant strains LF5, LF10, LF20, LF30, LF60 and LF240 fed on artificial diet containing 5.00, 10.00, 20.00, 30.00, 60.00 and 240.00 μg/g of Cry1Ac, respectively. Means with the different letters across tissues differed significantly (Tukey tests, *p* < 0.05).

Since the Bt-susceptible *H. armigera* larvae cannot survive on Bt-cotton, the resistant strain LF240 of *H. armigera* was used to analyze the tissue distribution of the Bt toxin in larvae when fed on Bt-cotton. ELISA results showed only trace amounts of the Cry1Ac toxin in the midgut, peritrophic membrane (PM) and its contents (hereafter as PM content), while no Cry1Ac was detected in the hemolymph, fat body or integument. The mean concentration of Cry1Ac toxin in Bt-cotton leaves (680.95 ± 125.80 ng/g) was lower than that in the artificial diets. Unexpectedly, we detected that the Cry1Ac levels in the PM content and midgut of larvae that were fed on cotton leaves were higher than those reared with artificial diets (Figure 2). This result is most likely due to the fact that the insects took much longer to develop to the 6th instar from neonates when fed on Bt-cotton leaves (20 days) than those fed on artificial diets (11 days), this may result in more toxins accumulated in the PM content and midgut. No Cry1Ac was detected in the hemolymph, fat body or integument, it could be explained that larvae with high resistance to Cry1Ac can prevent the toxin from passing through the midgut into other tissues based on different resistance mechanisms [3].

**Figure 3:**
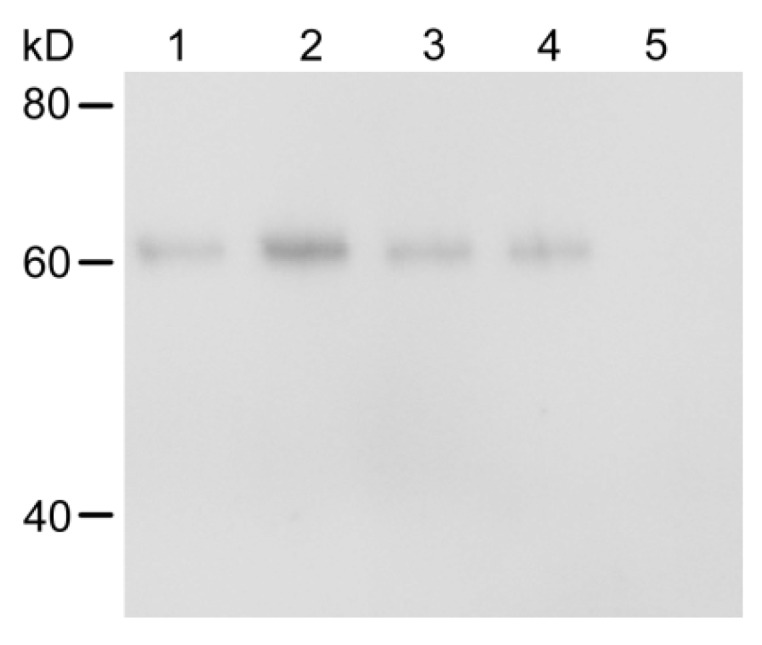
Western blot of 62-65 kDa fragment of Cry1Ac toxin in various tissues of larvae of Bt-resistant strain LF240 of *H. armigera* fed on an artificial diet containing 240 μg/g Cry1Ac toxin from the 1st to 6th instar. Lane 1, activated Cry1Ac toxin (5 ng); lane 2, peritrophic membrane and its contents (PM content, 50 μg); lane 3, midgut (1 mg); lane 4, hemolymph (1 mg); lane 5, fat body (1 mg).

In the subsequent experiment, larvae of strains LF5, LF10, LF20, LF30, LF60 and LF240 that had high resistance to Cry1Ac were assayed for Cry1Ac content in different tissues. Cry1Ac was only detected in the midgut and PM content when *H. armigera* fed on an artificial diet containing Cry1Ac at 5.00 μg/g of diet (Figure 2), similar to the results in the test with LF240 strain that fed on Bt-cotton leaves. Cry1Ac was detected in the hemolymph (0.28 ± 0.13 ng/g) when larvae were fed on the diet containing Cry1Ac at more than 10.00 μg/g of diet, and in the fat body (0.66 ± 0.01 ng/g) when the diet containing Cry1Ac at more than 30.00 μg/g of diet (Figure 2). The concentrations of the Cry1Ac toxin in the PM content were significantly higher than in other tissues (LF5: *p* = 0.0001; LF10: *p* = 0.0001; LF20: *p* = 0.0001; LF30: *p* = 0.0001; LF60: *p* = 0.0001; LF240: *p* = 0.0001). Although the Cry1Ac level in the midgut was higher than in the hemolymph, and fat body, the differences were not significant. Cry1Ac was not found in any integument samples. As expected, the concentration of the Cry1Ac in the four tissues increased as the amount of Cry1Ac increased in the diets (Figure 2). Western blot results showed that Cry1Ac toxin appeared to be present as the activated fragment with a molecular mass of 62-65 kDa and was only found in the PM content, midgut and hemolymph, not in the fat body, probably because the western blot is less sensitive than the ELISA assay (Figure 3).

### 2.3. Metabolic Status of Cry1Ac Toxin in H. armigera Larvae Tissues

**Figure 4:**
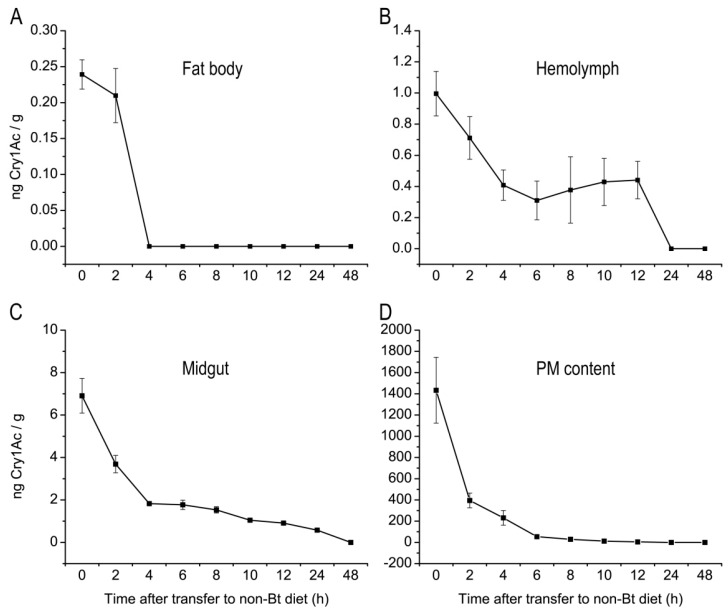
Mean concentration (± SE) of Cry1Ac toxin (ng Cry1Ac/g insects, *n* = 5) over time in larval tissues of *H. armigera* strain LF240 after transfer to non-Bt diet. Fat body (**A**); hemolymph (**B**); midgut (**C**); peritrophic membrane (PM) and its contents (PM content) (**D**).

Since Cry1Ac toxin was detected in the fat body, hemolymph, midgut and PM content of LF240 strain larvae, the metabolism of Cry1Ac in the four tissues was then examined. The content of Cry1Ac in the fat body of *H. armigera* larvae decreased quickly, and no Cry1Ac was detected within 4 h after the larvae were transferred to the non-Bt diet (Figure 4A). The decline in Cry1Ac contents in the hemolymph was slower than in the fat body, but the toxin had disappeared within 24 h after larvae were transferred to the non-Bt diet (Figure 4B). A similar tendency was found for the Cry1Ac level in the PM content; within 24 h of the transfer to non-Bt diet, the toxin was no longer detected. However, the toxin level in the PM content rapidly decreased in the first 6 h, and thereafter the toxin level declined at a slower rate (Figure 4D). The decrease of Cry1Ac in the midgut was lowest as shown in Figure 4C; the toxin was not completely gone until 48 h after transfer to the non-Bt diet. Overall, the amount of Cry1Ac in the fat body, hemolymph, midgut and PM content decreased over time after the switch to the non-Bt diet, and could not be detected after 48 h for all tissues.

## 3. Discussion

Cry1Ac toxin was only detected in *H. armigera* larvae, not in pupae and adults when fed on artificial diets containing Cry1Ac (Figure 1). These findings were consistent with the results from previous studies [20,21], that Bt toxin might be excreted with feces before pupation. In addition, the absence of Cry1Ac in pupae and adults suggested that Bt toxins are not transferred to natural enemies if they feed on or parasitize pupae, adults and eggs of *H. armigera*.

In previous reports, Cry toxin was found in the hemolymph, midgut and PM content of larvae when insects were reared with Bt plants [18,19]. The present study is the first to detect Cry1Ac in the fat body of lepidopteran larvae (Figure 2), likely because we used Bt resistant strains of *H. armigera*, that can survive from the 1st instar on high doses of Cry1Ac toxin, whereas susceptible insects can only survive feeding on Bt-crops for a few days. Cry1Ac in the PM content, midgut and hemolymph was shown to be present as the 62–65 kDa activated toxin fragment (Figure 3), which was demonstrated to be stable in in vitro activation studies and thermal treatment experiments [22,23,24]. Therefore, we speculated that Cry1Ac in the fat body of *H. armigera* larvae was still in the form of the activated toxin fragment.

The metabolic status of Cry1Ac in fat body, hemolymph, midgut and PM content of *H. armigera* larvae differed among the four tissues in the present study. The level of Bt toxin in the fat body decreased the most quickly (Figure 4A), perhaps because the Cry protein can interact with aminopeptidase N in the fat body of some lepidopteran insects [25,26,27], but the functional role of this enzyme as the Cry protein receptor has not yet been proven. Because the hemolymph can transport metabolites to an excretory organ such as the malpighian tubule, which maintains water, ion and acid/base balance and removes toxins and metabolic wastes [28,29], we speculated that Bt toxin in the hemolymph may be excreted via the malpighian tubule, so that Bt toxin was eliminated slower from the hemolymph (Figure 4B). In the PM content, Bt toxin was eliminated quickly at first, then slowed (Figure 4D), perhaps from binding of Bt toxin to the peritrophic membrane [30,31,32]. Bt toxin binds to specific receptors on the brush border membrane of the midgut cells, and part of the intact toxin is inserted into the membrane [11], which may thus explain why the midgut had the lowest decline in Cry1Ac toxin (Figure 4C).

Because the Cry1Ac toxin is present in larval tissues, the toxin might be ingested by natural enemies, that feed on or parasitize *H. armigera* larvae. Such is the case in *Harmonia axyridis*, after feeding on *Spodoptera frugiperda* larvae reared on Bt maize and in *Cotesia marginiventris* that parasitized *S. littoralis* larvae that had fed on Bt maize [20,33]. Moreover, previous studies have shown that the content of Bt toxin differs in herbivores that feed on Bt crops [34,35]; thus natural enemies of the herbivores are exposed to different doses of Bt toxin, resulting in diverse impacts on both predators or parasitoids. The negative effects of Bt crops have decreased the population of predators and their predation of insects had not been reported [36]. However, negative impacts of Bt crops on the fitness of parasitic natural enemies had been reported [20]. Therefore, this study of the Bt toxin in larval tissues may lead to a better understanding of the transfer of Bt toxin among organisms as well as its ecological toxicology in plant–herbivore–natural enemy food webs.

## 4. Conclusions

In summary, we detected the Cry1Ac toxin in *H. armigera* larvae, but not in pupae and adults, which suggested that the toxin was excreted with feces before pupation and that Bt toxins are not transferred to natural enemies that might feed on or parasitize pupae, adults and eggs of *H. armigera*. Furthermore, we found that the Cry1Ac toxin could be detected in the hemolymph of the 6th instar larvae when fed on an artificial diet containing more than 10.00 μg/g of Cry1Ac, and in the fat body when the artificial diets contained more than 30.00 μg/g of Cry1Ac, but the toxin was not present in the integument of *H. armigera*. Moreover, the content of Cry1Ac toxin in the four tissues increased as the amount of the Cry1Ac in the diets increased. Finally, the Cry1Ac toxin was decreased rapidly in the fat body but decreased slower in the hemolymph, peritrophic membrane and its contents. Metabolism of the toxin was slowest in the midgut, but in all cases the toxin was degraded completely within 48 h of the insects being removed from the Bt toxin diets.

## 5. Materials and Methods

### 5.1. Insect Culture

Seven laboratory strains of *H. armigera*: Bt-susceptible strain LFS (Langfang susceptible) and Bt-resistant strains LF5, LF10, LF20, LF30, LF60 and LF240 were used in this series of experiments. All the strains were reared on artificial diets with or without Cry1Ac at 27 ± 2 °C, 75% ± 10% relative humidity (RH) and a photoperiod of 14:10 (L:D) h. Adult moths were provided with 10% sugar and 2% vitamin complex.

The Bt-susceptible strain LFS was originally collected in 1998 from a field population in Langfang, Hebei Province, China and has since been maintained on an artificial diet without exposure to Cry1Ac or any insecticides [37,38,39]. A substrain of LFS was selected and allowed to feed on the artificial diet containing 5.00 μg/g of Cry1Ac from F_39_. When this substrain was adapted to 5.00 μg/g of Cry1Ac (mortality < 20%), it was further separated into two substrains. One was named as LF5, which was selected on diet containing 5.00 μg/g of Cry1Ac since then. The other was selected on a diet containing 10.00 μg/g of Cry1Ac from F_46_ until it was adapted to that level of Cry1Ac. It was then further separated into two substrains, one was named as LF10, which was selected on a diet containing 10.00 μg/g of Cry1Ac since then, the other was used to generate LF20, LF30, LF60 and LF240 on a diet with 20.00, 30.00, 60.00 and 240.00 μg/g of Cry1Ac, respectively. For this study, LF5, LF10, LF20, LF30, LF60 and LF240 had been selected for 160, 160, 100, 79, 77 and 23 generations on their corresponding Cry1Ac diets, generating a resistance range of 100–4000-fold greater than in the susceptible strain [39,40,41].

### 5.2. Quantification of Cry1Ac in H. armigera Body at Different Developmental Stages

The level of Cry1Ac toxin was quantified at different developmental stages in the body of Bt-resistant strains LF5, LF30, LF60 and LF240 of *H. armigera* were fed on artificial-diet containing selected doses of Cry1Ac toxin. Susceptible strain LFS was reared on the pure diet containing non-Bt protein used as a negative control. The Bt toxin in the 6th instar larvae (PM content included), pupae and adults was quantified using the ELISA assay described later.

### 5.3. Quantification of Cry1Ac in H. armigera Larval Tissues

To quantify the concentration of Cry1Ac in different larval tissues, leaves of Nucotn33B cotton, which expresses the Cry1Ac toxin in the whole plant, were used to rear the neonates retrieved from a Bt-resistant strain LF240, which can survive on Bt-cotton leaves without dying. The Bt cotton variety was used since it has been widely cultivated in China. Twenty days later, when the insects reached the 6th instar larvae, they were collected for dissection. To increase the exposure level of *H. armigera* larvae to Cry1Ac toxin, in the following tests, larvae of the resistant strains LF5, LF10, LF20, LF30, LF60 and LF240 were allowed to feed on Cry1Ac toxin at 5.00, 10.00, 20.00, 30.00, 60.00 and 240.00 μg/g, respectively, in the artificial diets starting with the 1st instar. Likewise, after 11 days feeding, when the insects reached the 6th instar, they were sampled and used to quantify the concentrations of the Cry1Ac toxin in the integument, fat body, hemolymph, midgut and PM content.

### 5.4. Metabolism of Cry1Ac in H. armigera Larval Tissues

For investigating the metabolism of Cry1Ac toxin in the fat body, hemolymph, midgut and PM content after the insects were removed from the Bt toxin, larvae of Bt-resistant strain LF240 were fed an artificial diet containing Cry1Ac toxin at 240 μg/g from the 1st instar to the early 5th instar, which were then transferred to the non-Bt diet. During the first 12 h, we excised the different tissues form insects every 2 h, then at 24 h and 48 h.

### 5.5. Tissue Collection

Larvae were washed with a saline solution (0.15 M NaCl) thoroughly before dissection to avoid diet contamination. Hemolymph was collected from alive larvae by cutting a proleg. The larvae were dissected from a side of the body carefully to avoid injuring the gut, then the gut was excised from the body. The peritrophic membrane and its contents (PM content) were pulled from the gut using ophthalmic forceps. The midgut was cut from the gut. The fat body was scraped from the integument of the larvae. The midgut, fat body and integument samples were separately washed thoroughly with precooled saline solution. Six replicates were prepared for each tissue, and each one was obtained from five different larvae and then pooled together.

### 5.6. Quantitative Enzyme-Linked Immunosorbent Assay (ELISA)

Levels of extracted Cry1Ac in insect samples and cotton leaves were measured using a Cry1Ab/Cry1Ac QualiPlate kit from EnviroLogix (Portland, ME, USA). To extract the Cry1Ac protein, the extraction buffer (phosphate-buffered saline with 0.55% Tween 20) was added to the samples (1:10 g sample to mL buffer) in the tube along with six 3.18-mm-diameter chrome steel balls (MP Biomedicals. Santa Ana, CA, USA) and were ground in a mixer mill MM400 (Retsch, Haan, Germany) for 5 min at 30 Hz/s. The extract was then shaken on platform shaker for 2 h at 4 °C and centrifuged at 10,000 rpm for 10 min; the supernatants were then used for ELISA detection. The extracts from larvae, PM content and Bt-cotton samples were serially diluted for the assay. A standard curve was generated using Cry1Ac protein (EnviroLogix) at 0, 0.0156, 0.0312, 0.0625, 0.125, 0.25, 0.5 and 1.0 ng/mL. The detection limit of the Cry1Ab/Cry1Ac QualiPlate kit was determined to be 0.0156 ng/mL on the basis of the optical density of the diluted standard Cry protein solution. The ELISA was done as directed in the kit instructions.

### 5.7. Western Blots

Western blots were used to determine the form of the Cry1Ac toxin in different tissues of *H. armigera* using a Cry1Ac monoclonal antibody (Fitzgerald, Boston, MA, USA). Tissues were excised from Bt-resistant strain LF240 larvae that had fed from the 1st instar to the 6th instar on the artificial diet containing Cry1Ac toxin at 240 μg/g. The protein was extracted as for the ELISA assay. For the Western blot, 50 μg total protein of the PM content sample and 1 mg total protein of the midgut, hemolymph and fat body samples were separated by SDS-PAGE with a 10% polyacrylamide gel, then electro-transferred to a PVDF membrane (Millipore, Billerica, MA, USA). The blot was blocked with Tris-buffered saline with Tween 20 (TBST, 1% (*v*/*v*) Tween 20 in 50 mM Tris-HCl, pH 7.5, and 150 mM NaCl) containing 5% (*w*/*v*) nonfat milk for 2 h at room temperature. The blot was washed three times with TBST buffer for 10 min, incubated with anti-Cry1Ac antibody (1:3000 dilution) overnight at room temperature, and washed described already. The membrane was then incubated with fluorescent secondary antibody 1:5000 dilution (Earthox, San Francisco, CA, USA) for 1 h and washed three times with TBST. Finally, the Bt bands were visualized using the Odyssey system (LI-COR, Lincoln, NE, USA).

### 5.8. Data Analysis

The amount of Cry1Ac toxin was compared among the various tissues of *H. armigera* and at different developmental stages using a one-way analysis of variance with a post hoc Tukey test in Statistica 6 (Statistca, SAS Institute Inc., Cary, NC, USA). A probability level of *p* < 0.05 was considered as statistically significant.
